# Evaluation of force generation mechanisms in natural, passive hydraulic actuators

**DOI:** 10.1038/srep18105

**Published:** 2016-01-04

**Authors:** A. Le Duigou, M. Castro

**Affiliations:** 1 Polymer and Composite, European University of Brittany (UEB), LIMATB-UBS, Lorient, France; 2 Smart Plastics Group, European University of Brittany (UEB), LIMATB-UBS, Lorient, France

## Abstract

Pine cones are well known natural actuators that can move their scales upon humidity gradient. The mechanism manifests itself through a displacement easily observable by the naked eye, but coupled with stress generation. In ancient Egypt, wooden wedges were used to break soft blocks of stone by the generated swelling stress. The purpose of the present study is to evaluate the ability of pine cone scales to generate forces while being wetted. In our experiments, a blocking force of around 3N is measured depending on the position on the pine cone where the scales are extracted. A fairly good agreement is obtained when theoretical results based on bimetallic strip systems are compared with experimental data, even if overestimation is observed arising from the input data considered for dry tissues. Inspired by a simplified pine cone microstructure, a biocomposite analogue is manufactured and tested. Although an adequate blocking force can be generated, it has a lower value compared to natural pine cones which benefit from optimized swelling tissue content and interfacial bond strength between them. This study provides new insights to understand the generation of force by pine cones as well as to develop novel biocomposite functionalities.

Plant movement can be very fast when the actuation principle is based on elastic instabilities^1^. However most of plant motion is rather slow as a consequence of cell-wall swelling/shrinking due to fluid transport^1^,^2^,^3^. In the latter case, a hierarchical plant tissue architecture constitued of single cells with a composite structure, i.e. stiff cellulose fibrils oriented in a swellable matrix^4^, is required to generate anisotropic swelling. When the plant tissues are strongly bonded together in a bilayer architecture, a complex mechanical response is generated that can involve folding, curling, twisting or bending.Natural actuators such as pine cone^5^,^6^,^7^ and wheat awn^8^ represent a source of inspiration for the development of humidity-driven bending or twisting actuators^7^,^9^,^10^,^11^,^12^ especially with synthetic materials. Recent studies have shown that the principle of natural actuators, based on a bilayer, can be used to develop self-shaping wood and plant fibre-based biocomposite actuators^13^,^14^ making use of the swelling ability of plant tissue such as vegetal fibres.

The swelling of wood tissues can also be converted into force. Indeed, in ancient Egypt, water saturated wooden wedges were used to break soft blocks of stone. Some studies have measured a restraint swelling stress of about 1.2 MPa for wetted spruce in the tangential direction^15^.

A recent trend in the development of bilayer synthetic actuators is to convert strain energy induced by differential swelling into force. Recently, Chen *et al.*^16^ have shown the potential of *Bacillus* spores to create water-responsive materials with high energy density leading to a mechanical response, as well as Mu *et al.*^17^ who reported fast, powerful and tunable actuation of graphene-based films being sensitive to moisture, heat and light.

The first objective of this study is to evaluate the ability of a natural actuator (i.e. pine cone) to convert the developed bending strain into a blocking force. Then, biocomposite analogues (e.g. vegetal fibres reinforced polymer composite) are manufactured inspired by the structure of pine cone tissues and making use of the anisotropic swelling of plant tissues. Several parameters such as fibre content and interfacial bonding strength are tested to improve our understanding of force generation mechanisms in natural actuators and also to develop novel biocomposite functionalities and applications.

## Results

### How much force can be developed by a pine cone?

**Figure 1:**
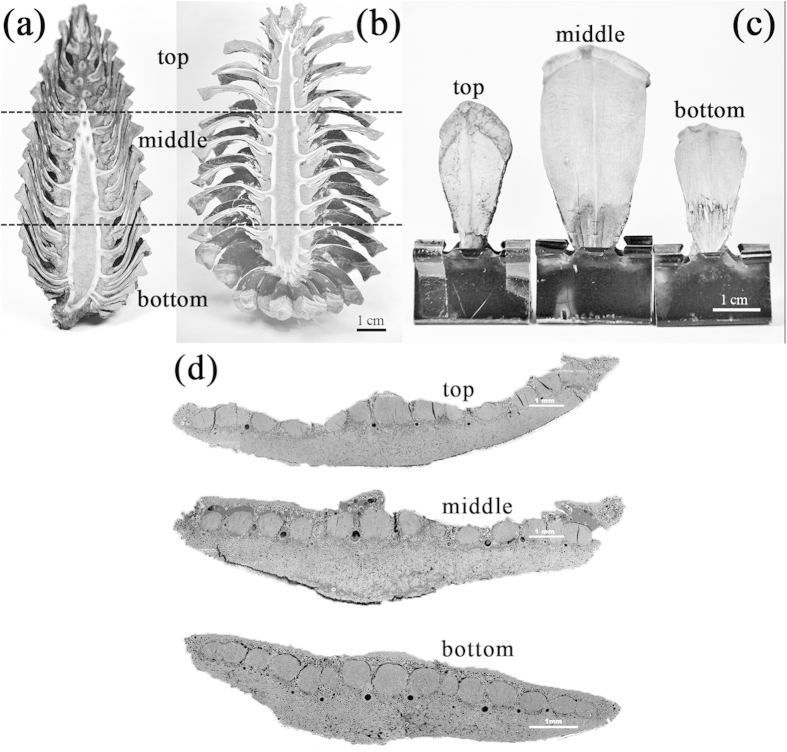
Cross-section of pine cone and scale geometry (Credit photo Le Duigou/Castro). (**a**) A pine cone in wet state and (**b**) in dry state. Female pine cones exhibit folding of their scales when dried to release seeds. Scale geometry depending of the location along the pine cone in the dry state (**c**). Depending on their location within pine cone, scales show different size and shape. The scale from the middle part is the largest and widest (see table 1) followed by top counterparts. Bilayer microstructure of pine cone in dry state (**d**). Scales show a bilayer microstructure whatever their locations, combining sclerenchyma fibre (around 35%) and sclerids (around 65%). However, these ratios change depending on the location of scale along the stem. Scale bars in (**a–c**) and (**d**) indicate 1 cm and 1 mm, respectively.

While wetting, pine cones close their scales (Fig 1a) basically to prevent seeds from shedding under humid weather^18^. Depending on their position on the pine cone axis (Fig. 1a,b), scales exhibit different geometry with larger size for middle scales (Fig 1c,d).

However their position does not influence the overall microstructure even if shape-induced curvature by seeds can be observed on the edge of the scale located in the middle and top parts of the pine cone (Fig. 1c). Pine cone scales always show a bilayer architecture. The first layer is composed of tissue that provides stiffness (sclerenchyma fibre with low MFA around 0°) while the other layer generates high strain (sclerids with high MFA around 90°)^4^ with ratios which depend on their locations (Table 1).

**Figure 2:**
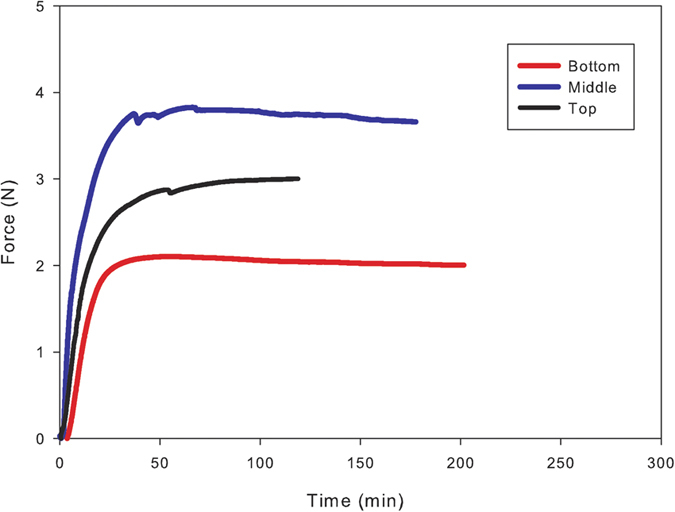
Typical blocking force generated by scales during wetting as a function of time for different locations along the pine cone. Force increases very quickly during the first hour of immersion. Then generated force is stabilized. Scale from middle of the pine cone exhibits higher force potential.

Force measurements are performed on the extracted cone scales during wetting by preventing their movements with a rigid needle. Measurements are performed as a function of their location along the stem (bottom, middle and top part). Once immersed in water, cone scales generate a blocking force due to differential swelling between each tissue (Fig. 2). This mechanism is similar to those of bending and is now relatively well understood and has been well-described in the literature^5^,^6^,^7^. Blocking forces exhibit a rapid linear increase from 0 to about 30 min and then level off to a plateau after 50 minutes whatever the position of extraction of the scales (Fig. 2). No considerable force reduction is observed after the maximum force value is attained (on the considered time scale). This means that mechanosorptive creep is negligible and thus the hygroscopic load transfer between each layer (Fig. 2) is of high level and can withstand water degradation.

The response time of the pine cone, i.e. the force generation rate (slope of the force versus time curve) is higher for scales from the middle location (Table 1) even if the time to reach maximum blocking force is similar.

Blocking force and moment values are reported in Table 1, illustrating the role of pine cones as a source of inspiration for developing not only water-induced bending actuators but also force-generation actuators. Indeed, pine cone scales are able to generate a blocking force ranging from 2.2 to 3.6 N during wetting depending on their location on the pine cone. Such values are slightly lower than *Selaginella lepidophylla* during drying^19^. This comparison should be taken with caution as this latter is thinner than pine cone scale and that experiments have been achieved during dehydratation which omit the plasticising effect of water. This force is however drastically higher than values obtained for synthetic actuators such as: 3–7 mN for buckypaper/Nafion® membrane^20^, around 50 mN for IPMC actuator^21^ and around 40 mN for buckypaper-supported ionic liquid membrane^22^. Comparisons should be drawn with caution since electro-active synthetic actuators exhibit a very rapid active response while pine cones have a passive slow behaviour. Knowing the microstructure of cone scales, i.e. thickness and properties of each tissue component and their respective content (table 1), we can apply bi-metallic theory^23^ to estimate the blocking force, F, developed by a strip tightened at one end and having a pin at the other end (Eq. 1).


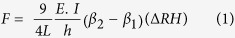


With *L* the bending length span, *E* the Young modulus of the pine cone scale in the dry state (which is the combination of stiffness of each component). Dry state is chosen here since, to our knowledge, no published data exists on wetted tissues because their dissection is particularly difficult^5^. The moment of inertia, *I*, of a scale is assumed to be equivalent to a rectangular beam of constant width, *b*, and the thickness of a rectangular beam is taken as equal to 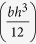. *β* is the hygroexpansion coefficient of each tissue. Indices 1 and 2 are assigned here to the sclerids and fibre sclerenchyma, respectively.

**Figure 3:**
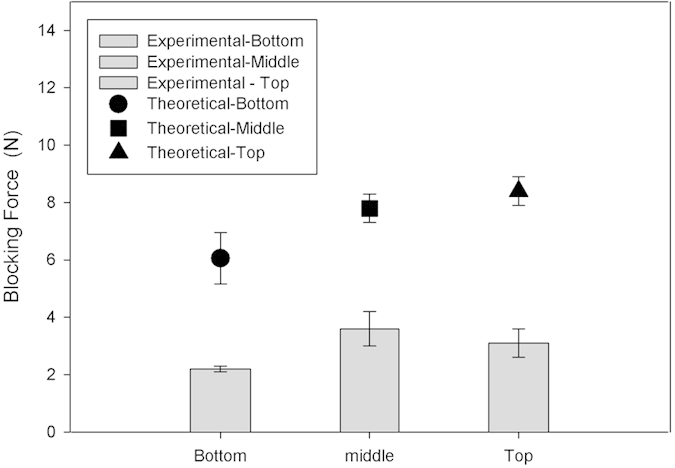
Experimental force generated according to the location of the pine cone scale compared to theoretical predictions. Theoretical prediction evidences a difference with experimental measurements. The discrepancy is likely due to the hypothesis of using the dry properties of pine cone tissue from literature, instead of wet counterparts, that would lead to a 50% reduction of Young’s modulus.

The experimental blocking force of pine cone scales according to their location is thus compared with the theoretical prediction for bi-metallic strips (Fig. 3).

The bi-metallic model clearly appears to overestimate the experimental blocking force, even if values remains in the same range. Considering our hypothesis of using the dry properties of pine cone tissue from published work^5^ instead of wet counterparts and that all tissues have a similar chemical composition, we need to take into account a 50% reduction of Young’s modulus for dry tissue to fit the experimental data with the model.

During wetting, a lowering of Young’s modulus is highly likely due to plastification of pine cone scales in view of the observations of Joffre *et al.*^24^ on wood tracheid polysaccharides (hemicellulose and lignin) . Pine cone scale tissues are composed of 20% v_f_ cellulose with properties that are independent of moisture^24^. The other components (around 80%), including lignin, hemicellulose and pectins with an unknown distribution^5^ participate in load bearing and their stiffness falls after wetting, respectively, from 11 GPa to around 1 GPa (>100% loss for hemicellulose), and from 1.5 GPa to 0.7 GPa (>50% loss for lignin)^24^. Therefore such difference between theoretical and experimental values could be solved by taking into account input data for wet tissues.

To sum up, pine cone scales exhibit a versatile response to moisture gradient, showing a capacity for both bending and force actuation. This latter ability could be useful for pine cone to open against exogen factors such as wind, neighboring branches, other pine cone or when it has fallen on the ground (to lift its own weight)… that could hinder the opening. In addition high blocking force is also required for seeds protection to prevent unwanted opening (by animals for instance…).

To understand pine cone force actuation in more detail, a biocomposite analogue is manufactured and tested which actuates through a similar water-driven process. Two material parameters are analysed here, swelling fibre content and interfacial bonding strength.

### Biocomposite analogue to understand the generation of force by pine cones

#### Biocomposite analogue design and microstructure

Bast fibres such as flax fibres have a multi-scale structure with mechanical properties strongly linked to their S2 cell-wall composition^25^. S2 cell-walls of flax fibres have a small MFA, around 10° 26, which leads to anisotropic hygro-elastic properties.

In a similar way, the cellulose microfibril angle controls the swelling of single fibres and the response of natural actuators (pine cone, wheat awn, etc.)^4^. Anisotropic swelling of flax fibres and their orientation within the analogue is assumed to control the response.

**Figure 4:**
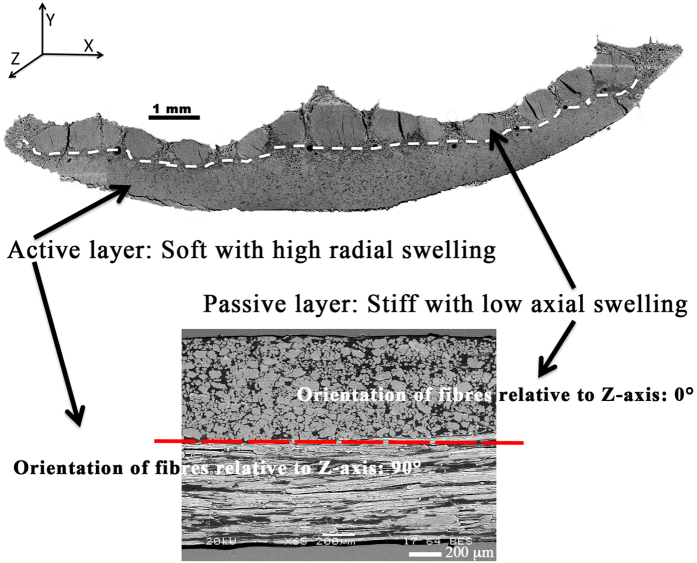
Description of biocomposite analogue, based on pine cone scale microstructure. (**a**) Cross section SEM image of a pine cone scale highlighting a bilayer structure. (**b**) Flax fibres are strongly anisotropic and are used as a swelling agent in our analogue. A bilayer composite is manufactured with orientations of flax fibres (vf=60%) within each layer set at 0° and 90°, respectively mimicking the sclerenchyma fibres (passive layer with stiffness and low axial swelling) and sclerids (active layer with softness and high radial swelling) of a pine cone. Flax fibre content varies from 17, 40 to 60%.

In this study, we manufactured water-responsive biocomposite analogues with a bilayer structure inspired by those pine cone (Fig. 4). Unlike the previous biocomposite analogue^14^ which was composed of a passive layer achieved with a highly apolar polymer, i.e. polypropylene, here each layer is composed of flax fibres embedded in the same polymer matrix. Mechanical behavior and properties (E = 1000 ± 53 MPa) of Polypropylene is assumed to be independent of water effect (for the time range and temperature considered).

The orientations of flax fibres are set at 0° and 90° in each layer, respectively mimicking the sclerenchyma fibres and sclerids of a pine cone (Fig. 4).

The pine cone scale microstructure is also described in terms of a naturally optimized volume ratio between the sclerenchyma fibres and sclerids (around 70% of sclerids (swelling tissue), see Table 1) and between the components within each tissue to produce bending and force actuation. Indeed, the tissues, sclerenchyma fibres and sclerids are composed of tubular bundles of single fibres tightly bonded together^27^ by middle lamellae incorporating a very high content of single fibres. Clearly, due to the manufacturing and wetting processes, the biocomposite analogue cannot attain such a high fibre content. Therefore, the blocking force of the biocomposite analogue is quantified as a function of fibre content (17, 40 and 60% vol) and varying interfacial bonding strength due to polymer matrix grafting to investigate the actuation mechanism.

#### Force generated by the biocomposite analogue

**Figure 5:**
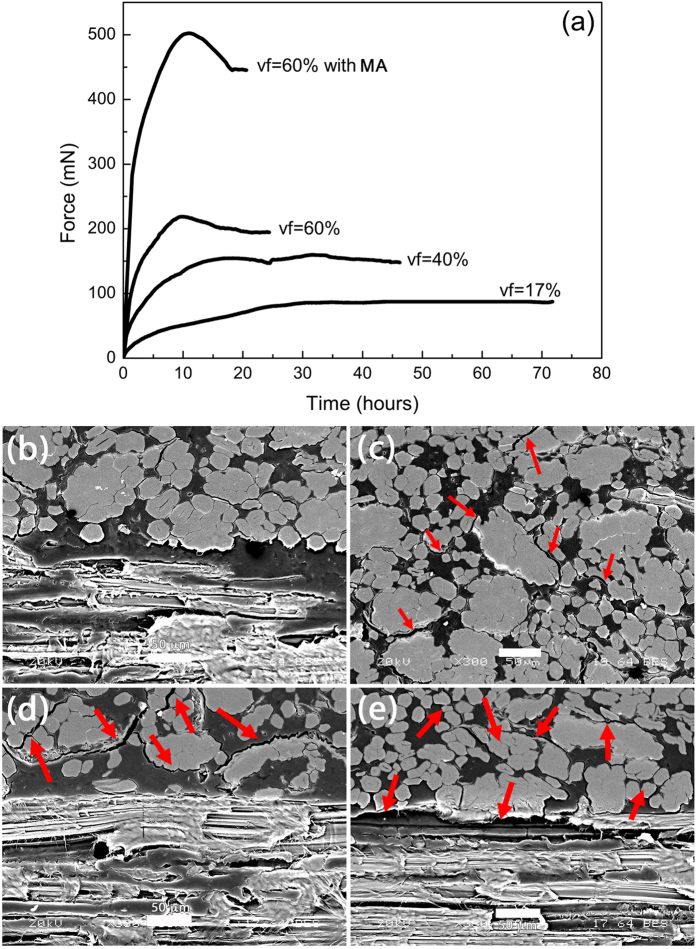
(**a**) Typical blocking force vs. immersion time behaviour for different fibre volume content and fibre/matrix interface modification via maleic anhydride (MA). A two step behavior with a large increase of force for short immersion time is observed for all the analogues, followed by a loss of linearity. High fibre content induces a slight reduction of the force after the maximal value is obtained. SEM observations. (**b**) Interlaminar area vf=60% MA non-tested : no damage observed in the interlaminar zone. (**c**) Interfacial area vf=60% MA non-tested : damage of fibre bundle (see red arrows). (**d**) Interlaminar area vf=60% MA tested: no delamination in the 0-90° area, but fibre/bundle and fibre/matrix decohesion as well as bundle division (see red arrows) (**e**) Interlaminar area vf=60% tested: delamination in the 0-90° area + fibre/bundle and fibre/matrix decohesion and bundle division (see red arrows). Scale bars in (**b–e**) indicate 50 μm.

Once immersed in water and tightly blocked at one end, biocomposite analogues are able to generate force (Fig. 5) as pine cone scales do. Figure 5 shows the typical actuating behaviour of biocomposite analogues as a function of fibre content. A two-step behavior is observed, with a large increase for short immersion time (<10 h for 60% vol flax biocomposite) and then a loss of linearity, especially for fibre contents above 17%. High fibre content biocomposite analogues exhibit a slight reduction of the force after the maximum value is reached, which could be attributed to mecano sorptive degradation as indicated by SEM images (Fig. 5b–e), with splitting of damaged bundles, debonding of the fibre/matrix interface and also delamination of the bilayer for biocomposites with 60% fibre content. Polypropylene matrix yielding as well as plant fibres creep could also occur due to their visco-elastic behavior. Such degradations have already been observed when bending of PP/flax actuators is possible^14^. However, bilayer delaminating is limited if the passive layer in manufactured with neat PP.

**Figure 6:**
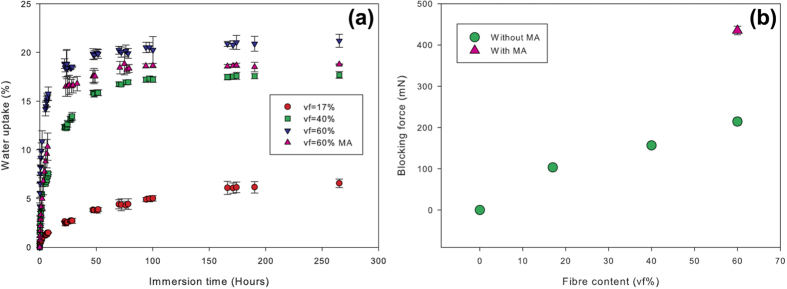
(**a**) Water uptake as a function of fibre content and interfacial modification. Increasing fibre content accelerates the water sorption kinetic but also the level of water uptake at saturation. Improvement of fibre/matrix interface bond, with maleic anhydride (MA), reduces water absorption kinetic and overall water content at saturation. (**b**) Average maximum blocking force as a function of different fibre volume content and fibre/matrix interface modification. Blocking force increases linearly with fibre content. Improvement of fibre/matrix interface (with maleic anhydride) increase the blocking force by 100%.

The increase of fibre content in each layer of the biocomposite analogue leads to a shortening of their response time (Fig. 5a), from 3.1 to 11.1 mN/min for 17 to 60% vf, due to the hydrophilic nature of flax fibres. Indeed, the higher the fibre content, the faster the rate of increase of water uptake and the higher the maximum value of water absorption (from 6.1% to 20.8% for 17 and 60% fibre volume fraction, respectively). At the time required to obtain the maximum force (around 10 hrs according to Fig. 5a), we can observe that maximal water uptake is not attained on gravimetric samples that have not undergone force tests (Fig. 6a). Again, a similar offset has been observed on biocomposite analogue with programmable bending^14^, between curvature and water uptake. Thus water uptake generates swelling stress and mechano sorptive creep degradation which in turn modifies sorption mechanism^28^. Without interfacial improvement, the maximum blocking force is linearly controlled by swelling fibre content up to value of 214.3 ± 8.9 mN for the loaded sample with vf = 60% vol. (Fig. 6b). Indeed, increasing flax fibre content also linearly enhances the transverse swelling of our analogue from 1.2% for vf = 17% vol. to 4.4% for vf = 60% vol. However, flax fibres exhibit transverse stress-free radial swelling β_T_ of around 25%^29^. Following a simple mixing law and assuming β_PP_ = 0%, flax fibres within the PP matrix show dramatically lower values of β_T_ around 7.1 ± 0.5%. This corroborates the observations of Joffre *et al.*^30^. Therefore, it is crucially important to understand the swelling mechanism of plant fibres to improve the performance of the prepared biocomposite analogue.

Water content and swelling ability are not the only parameters controlling the response of the biocomposite analogue and thus the pine cone. Indeed, improving the interfacial bond strength by using grafted PP-g-MA (while keeping the fibre content constant) reduces the total amount of water uptake to around 18.3%wt. and slows down the water uptake rate (Fig. 6a). However, the blocking force rate for the MA-modified biocomposite appears to be faster than the other tested materials, 21.5 mN/min (Fig. 5a), and the maximum blocking force is drastically improved (+100%), e.g. 435 ± 10.2 mN. In addition, SEM images (Fig. 5b–d) show that grafting PP matrix with MA reduces mechanosorptive damages such as delamination between layers as well as between flax fibre and matrix. Van der Waals interactions as well as adhesive pressure coming form thermal residual stress are responsible for a major part of interfacial shear strength but are water sensitive. Modifying PP with maleic anhydride promotes stronger interactions, i.e. acid/base interactions in the interfacial area. Paradoxically, hygroscopic stresses contribute to actuation phenomenon but also induce the biocomposite loss of actuation properties.

Force actuation of our flax composite analogue is lower than those of wood bilayer actuator recently published^13^ (1–1.5N) but presents the advantages of being manufactured by industrial process that enables high shapability as well as taking benefit of by-products with low economical value.

## Discussion

By comparing the response of biocomposite analogues to pine cone scales, it is clear that the maximum force and the time required to reach the maximum force are far below the values obtained for pine cones (around 2–4 N). Possible explanations are as follows:

- A low content of swelling fibres in the analogue compared to pine cone scales. Indeed, pine cone scales are composed of 62–69% by volume of swelling tissue (Fig. 1d), while biocomposite analogues are composed of 50%vol. swelling layer (90°), which itself contains, in the best possible case, 60%vol. of swelling fibre. Thus, 30% of the total volume of the biocomposite is composed of swelling fibres.

- Despite the use of a grafting agent, biocomposite analogues suffer from weak interfacial interactions and marked creep mechano-sorptive degradation. A tight interface allows transfer of the hygroscopic load from one component of the pine cone scale to another, leading to bending while wetting^27^. This interface appears more durable against water degradation, since several drying-wetting cycles occur during the lifespan of a pine cone (from its growth to the ripening and even after its fall on the ground).

- One limit to the speed of the movement is determined by the time taken to transport the water across the tissue^1^. In addition to hydroxyl groups located on polar polysaccharides, the microstructure of a pine cone is composed of tracheid fibres with a lumen at their centre, therefore creating high porosity which promotes water transport and speeding up of the mechanism^2^. Water transport within the analogue is due solely to flax fibres.

- Pine cones exhibit a biochemical composition that is very different from flax fibres, the latter having a very low lignin content (a few %)^5^,^31^. Lignin could enhance durability against water as shown in the case of coir fibres as compared with flax^32^. Such a composition probably maintains greater stiffness compared to flax, whose stiffness may be affected by plastification^33^.

In summary, pine cone scales can generate blocking force of around 3N while being wetted that could be fairly well predicted by bimetallic theory if data from wet tissues are taken into account. Drawing inspiration from a simplified pine cone microstructure, biocomposite analogue manufacturing and testing have underlined that swelling fibre content and strong interfacial bond strength promote quick response with higher force. Although having lower blocking force value compared to natural pine cone scales whose efficiency have been naturally optimized during millions of years of evolution, the response of biocomposite analogues remains better than the synthetic counterparts^21^,^22^. As such, biocomposites offer a promising potential for developing autonomous building blocks or water driven force-generator devices based on inexepensive and local swelling plant tissues in developing countries.

## Methods

### Materials

Female pine cones from *Pinus pinaster* were collected, while still being attached to the tree, in Lorient (France) during June. Pine cones were first soaked in water at room temperature up to saturation to extract single scales for force measurements. Scales were manually removed from the bottom, middle and top part of the rachis. The cones were then kept at 50% RH and 23 °C for several days to reach equilibrium.

Bio-inspired biocomposites analogues were manufactured by hot pressing (190 °C during 8 min) unidirectional flax-fibre tapes (200 g/m^2^ and/or 50 g/m^2^), used as a swelling agent, which were then embedded in virgin polymer matrices of polypropylene film (PP) and compatibilized with maleic anhydride (MA) to improve interfacial bond strength^34^. The flax fibre content within the biocomposite analogues was set at 17, 40 and 60% by volume. Then biocomposites were machine–milled with specific geometry.

According to considerations previously taken into account for pine cone scales (active to passive thickness ratio, t_a_/t_p_; and total thickness, t_total_), the water-responsive biocomposites were manufactured with a similar overall thickness, i.e. 1.2 mm.

### Gravimetric analysis

Five samples (Length: 70 mm, width: 10 mm and thickness: 1.2 mm) were immersed in deionized water until constant weight is obtained. Samples were periodically removed to be weighed and characterized. The percentage gain at any time *t*, M_t_, was determined by Eq. (2):


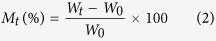


where W_t_ and W_0_ are the weight of sample after water exposure and the weight of dry material before immersion, respectively. The maximum moisture absorption, M∞, was calculated as the average value of several consecutive measurements. Desorption of the five saturated samples was measured under laboratory conditions, *i.e.* RH = 50% and T = 23 °C, by continuous recording of variations using a weighing device (10^−4^ g). All the results were averaged arithmetically.

### Swelling measurement

Three square-shape (50 × 50 mm^2^) biocomposite samples are cut out for each fibre content. Three lines are plotted longitudinally and transversally to the fibre orientation to ensure that swelling measurements are always performed at the same point. The results are then averaged arithmetically.

### Blocking force measurement

A home-made device is developed to measure the blocking force during sorption. Deterministic approach rather than stochastic is used here for measurement on pine cone scales. Similar approach has been successfully applied on vegetal fibre^35^. All the tests are performed on a single pine cone to minimize uncertainities arising from the variation when a set of pine cone is used. Thus five pine cone scales extracted from the bottom, middle and top part of the rachis are characterized.

**Figure 7:**
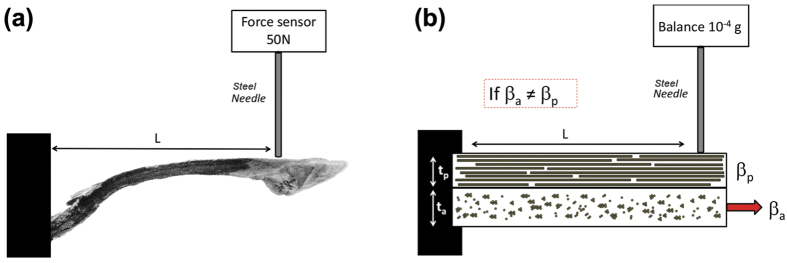
Diagrams showing principle of blocking force measurement on (**a**) pine cone scale and (**b**) bicomposite analogue of length L. β_a_ and β_p_ being the hygroexpansion coefficient of the active and passive layer, respectively. ta and tp being the thickness of the active and passive layer, respectively. Once immersed in water, the differential hygroexpansion coefficient of layers induces force generation.

Samples are clamped between metallic grips. A steel needle is set up in the tensile grips and then placed in contact with the tip of the pine cone scale, thus constraining its movement. A 50 N force sensor is used to measure the blocking force accurately (Fig. 7a). The results obtained are averaged arithmetically.

Five biocomposite analogues samples are also evaluated as force generators during the wetting process, but with the same 10^−4^ g balance (Denver SI14 instrument) than those used for gravimetric analysis. Biocomposite samples are clamped between metallic grips. A steel needle is screwed in under the weighing device and then put in contact with the tip of the biocomposite, thus constraining its movement (Fig. 7b).

### SEM observations

All the samples are sputter-coated with a thin layer of gold in an Edwards Sputter Coater, and analysed with a Jeol JSM 6460LV scanning electron microscope at 20 kV accelerating voltage to have a global observation of the scale microstructure as well as damage mechanism.

### Tissue analysis

Following pine cone scale observation, image analysis is performed using ImagJ software (National Institutes of Health, USA). The thickness and area of each tissue (sclerenchymafibre and sclerids) is determined on each sample (5 replications) and then averaged.

## Additional Information

**How to cite this article**: Le Duigou, A. and Castro, M. Evaluation of force generation mechanisms in natural, passive hydraulic actuators. *Sci. Rep.* **6**, 18105; doi: 10.1038/srep18105 (2016).

## Figures and Tables

**Table 1:** Geometrical parameters of scales according to location along the pine cone and blocking forces.

Location along cone axis	Top	Middle	Bottom
Average maximum thickness (mm)	1.3	1.5	1.1
Swelling tissue area (sclerids) (%)	65.5	69	62.5
Blocking force rate (N/min)	0.19	0.38	0.156
Blocking force (N)	3.1 ± 0.5	3.6 ± 0.6	2.2 ± 0.1
Blocking moment (N.mm)	80.5 ± 9.1	107.2 ± 20	42.0 ± 4.5
